# Brain nonapeptide and gonadal steroid responses to deprivation of heterosexual contact in the black molly

**DOI:** 10.1242/bio.20149597

**Published:** 2014-12-19

**Authors:** Ewa Kulczykowska, Hanna Kalamarz-Kubiak, Marta Nietrzeba, Magdalena Gozdowska

**Affiliations:** Department of Genetics and Marine Biotechnology, Institute of Oceanology of Polish Academy of Sciences, Powstańców Warszawy 55, 81-712 Sopot, Poland

**Keywords:** Arginine vasotocin, Isotocin, Sex steroids, Masculinization, Same-sex sexual behaviour, Black molly

## Abstract

Fish may respond to different social situations with changes in both physiology and behaviour. A unique feature of fish is that social interactions between males and females strongly affect the sexual characteristics of individuals. Here we provide the first insight into the endocrine background of two phenomena that occur in mono-sex groups of the black molly (*Poecilia sphenops*): masculinization in females and same-sex sexual behaviour, manifested by gonopodial displays towards same-sex tank mates and copulation attempts in males. In socially controlled situations, brain neurohormones impact phenotypic sex determination and sexual behaviour. Among these hormones are the nonapeptides arginine vasotocin (AVT) and isotocin (IT), counterparts of the well-known mammalian arginine vasopressin and oxytocin, respectively. To reveal potential hormone interactions, we measured the concentrations of bioactive AVT and IT in the brain, along with those of the sex steroids 17β-estradiol and 11-ketotestosterone in the gonads, of females, masculinized females, males displaying same-sex sexual behaviour and those who did not. These data were supplemented by morphological and histological analyses of the gonads. Correlations between brain nonapeptides and gonadal steroids strongly suggest a cross talk between hormonal systems. In the black molly, the masculinization process was associated with the production of brain AVT and gonadal steroids, whereas same-sex sexual behaviour involves both brain nonapeptides, but neither of the sex steroids. This study extends current knowledge of endocrine control of phenotypic sex and sexual behaviour in fish and for the first time links brain nonapeptides with the occurrence of male-male sexual behaviour in lower vertebrates.

## INTRODUCTION

Fish may respond to different social situations with changes in both physiology and behaviour. A unique feature of fish is that social interactions between males and females strongly affect the sexual characteristics of individuals (Devlin and Nagahama, 2002). Moreover, some teleosts, even in adulthood, spontaneously undergo phenotypic sex reversal in response to signals from the external environment (Godwin, 2010). A diversity of sexual patterns in fishes has been reviewed excellently by Sadovy de Mitcheson and Liu (Sadovy de Mitcheson and Liu, 2008). This lability in sex expression makes fish useful in studies of endocrine mechanisms underlying socially related changes of sexual phenotype and behaviour. The historically dominant view that sexual behaviour is under the control of sex steroids has recently shifted towards a paradigm in which brain neurotransmitters and neuromodulators play a decisive role in the determination of sexual phenotype and behaviour in the many fish species where sex change is under social control (Semsar and Godwin, 2003; Godwin, 2010). In socially controlled changes, brain neurohormones integrate and interpret social cues (Perry and Grober, 2003). There are several categories of signalling molecules in the brain that may be related to the sexual plasticity of fish, namely gonadotropin-releasing hormone (GnRH) peptides, monoamines (serotonin, noradrenaline, dopamine), and the nonapeptides arginine vasotocin (AVT) and isotocin (IT) (for review: Foran and Bass, 1999; Bass and Forlano, 2008; Godwin, 2010; Godwin and Thompson, 2012). AVT and IT, the teleostean equivalents of mammalian vasopressin and oxytocin, respectively (Acher and Chauvet, 1995), have attracted much attention due to their role in the regulation of social and reproductive behaviours (for review: Bass and Forlano, 2008; Godwin, 2010; Godwin and Thompson, 2012). More than 60 years ago Pickford (Pickford, 1952) demonstrated that neurohypophysial extracts induced a “spawning reflex” in hypophysectomized killifish (*Fundulus heteroclitus*) and later proposed AVT as an active component (Pickford and Strecker, 1977). Subsequent studies on AVT mRNA expression, AVT-immunoreactivity, the size of AVT-producing cells (for review: Godwin and Thompson, 2012) and AVT receptors in the brain (Semsar et al., 2001; Semsar and Godwin, 2004; Huffman et al., 2012; Lema et al., 2012; Soares et al., 2012; Oldfield et al., 2013) have revealed the contribution of AVT to social and reproductive behaviours in various fish species. In contrast, much less research has been dedicated to IT, which regulates paternal behaviour in a monogamous cichlid fish (O'Connell et al., 2012), courtship in the three-spined stickleback (*Gasterosteus aculeatus*) (Kleszczyńska et. al., 2012), social approach of males in goldfish (*Carassius auratus*) (Thompson and Walton, 2004) and vocal communication in the plainfin midshipman (*Porichthys notatus*) (Goodson and Bass, 2000). Recently, analyses of AVT and IT concentrations in the brains of several fish species have shown that these nonapeptides are related to specific phases of breeding or the social status of individuals (Ripley and Foran, 2010; Kleszczyńska et al., 2012; Sokołowska et al., 2013).

While the role of AVT in reproductive behaviour is well established in many fish species, its involvement in process of masculinization or sex change is less well characterized (Grober and Sunobe, 1996; Reavis and Grober, 1999; Godwin et al., 2000). Furthermore, in comparison with AVT, very little is known about the action of IT. Lastly, despite the fact that same-sex sexual behaviour has been described in many fish species (Morris, 1951; Bagemihl, 1999; Field and Waite, 2004; Tobler et al., 2005; Cureton et al., 2010, Bierbach et al., 2013), the endocrine background of this phenomenon has never been studied in fish or in other lower vertebrates. The present study is an attempt to fill these knowledge gaps. Previous studies have identified relationships between AVT and androgen levels, and sexual phenotypes in fish (see for example: Goodson and Bass, 2001; Semsar and Godwin, 2004; Oliveira et al., 2005; Aubin-Horth et al., 2007). The aim of the present study was to examine the response of female and male fish to deprivation of sexual partners by measuring its effect on the levels of nonapeptides in the brain and steroid levels in the gonads. We used a procedure to determine the concentration of free nonapeptides AVT and IT after their dissociation from non-covalent complexes. This was important because only this nonapeptide fraction binds to specific receptors to act as neurotransmitters and/or neuromodulators in the brain. This analytical procedure, which permits the measurement of bio-active nonapeptides AVT and IT at their site of action, has been used successfully, with slight modifications, in several fish species (Gozdowska et al., 2006; Kleszczyńska et al., 2012; Almeida et al., 2012; Kleszczyńska and Kulczykowska, 2013; Sokołowska et al., 2013; Martos-Sitcha et al., 2013). However, in small fishes such as black molly, a limitation of the procedure is that AVT and IT levels can only be analyzed in whole brains, and not in separate populations of neurones, and AVT mRNA expression in magnocellular and parvocellular neurones in the POA (preoptic area) appears to be regulated independently during sex change (Perry and Grober, 2003).

Several years of research on the black molly (*Poecilia sphenops*) conducted in our laboratory indicate that this well-known aquarium fish is a good model candidate for the present study. This species has several useful distinctive characteristics. It displays conspicuous sexual dimorphism: the males are smaller than the females and have a larger dorsal fin and an anal fin modified into an intromittent organ, the gonopodium. When kept in a mono-sex group, females undergo masculinization and males exhibit same-sex sexual behaviour, manifested by gonopodial displays towards same-sex tank mates and copulation attempts. These phenomena are triggered off in a reproducible manner under laboratory conditions. The masculinization process proceeds only in one direction, is irreversible and generates individuals with distinct features: they are the size of females, have well formed gonopodia and changed morphology of gonads. Moreover, they manifest male-like sexual behaviour with courting (gonopodium swinging, see later) and attempted copulation with females. On the other hand, males in male-only groups display male-to-male sex attraction, with behaviour equivalent to attempted copulation. In mixed sex groups, all males mate only with females. Conveniently, there is no social hierarchy within a group of black mollies, so social rank did not influence the results of this study (Backström and Winberg, 2009; Maruska et al., 2013).

Using the black molly model, we have compared the brain concentrations of AVT and IT, and the gonadal concentrations of 17β-estradiol (E2) and 11-ketotestosterone (11-KT) in females, masculinized females, males that exhibited gonopodial displays towards tank mates and copulation attempts, and those that did not. Any relationships between nonapeptides and steroids were verified statistically. We also performed morphological and histological analyses of gonads. Finally, we considered the nature of the signal triggering masculinization and same-sex sexual behaviour in the black molly. Our findings extend current knowledge of the endocrine background of phenotypic sex and sexual behaviour in fish, and for the first time link brain nonapeptides with the occurrence of same-sex sexual behaviour.

## RESULTS

**Fig. 1.**
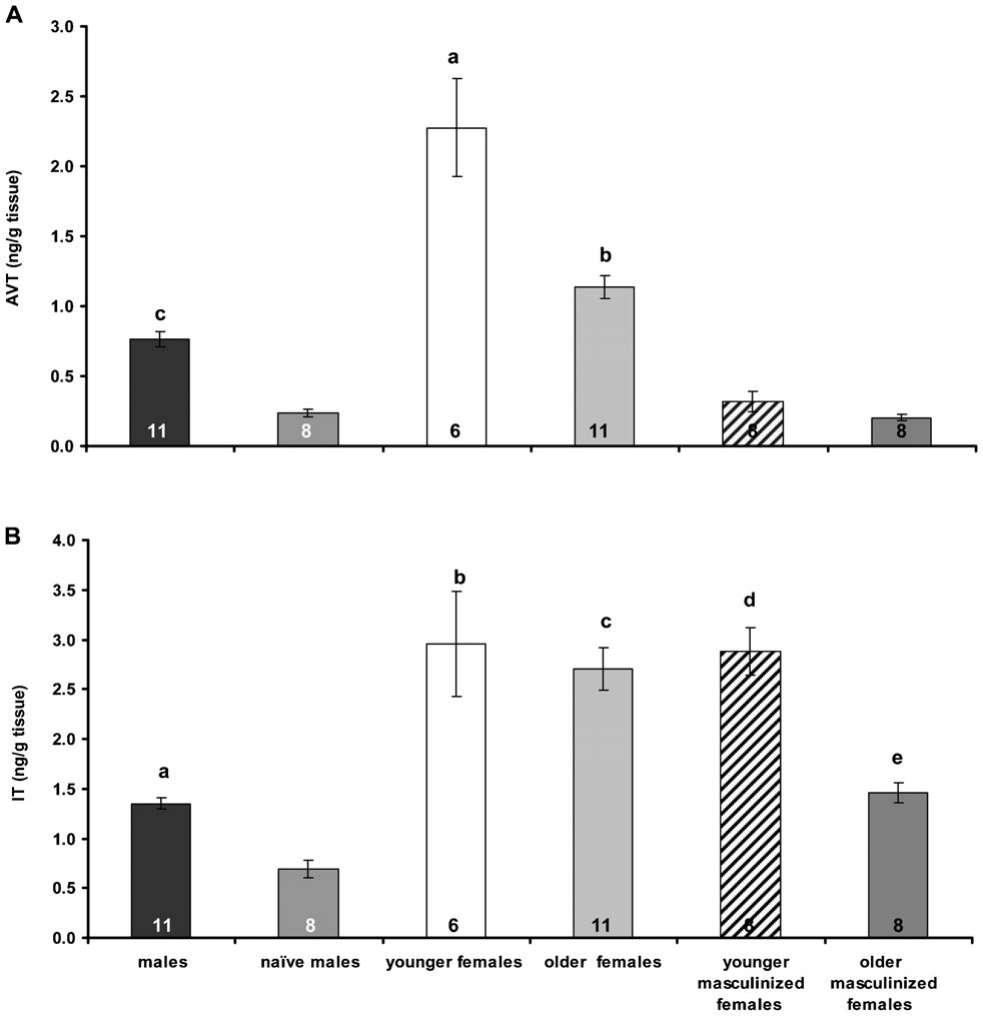
Arginine vasotocin (A) and isotocin (B) in brains of males, naïve males, younger and older females and masculinized females of *Poecilia sphenops*. Values are presented as means ± SEM. Statistical differences are indicated as: (A) a: *P*<0.001 vs the others, b: *P*<0.001 vs naïve males, younger and older masculinized females, c: *P*<0.05 vs naïve males and older masculinized females. (B) a: *P*<0.05 vs naïve males, b: *P*<0.001 vs males and naïve males, c: *P*<0.001 vs males, naïve males and older masculinized females, d: *P*<0.01 vs naïve males, younger and older females.

The brain AVT (Fig. 1A) and IT (Fig. 1B) concentrations were determined in different groups of the model fish black molly: younger and older females and masculinized females, males that displayed same-sex mating (called naïve males) and those that did not. Brain AVT levels were lowest in masculinized females and naïve males. Brain IT levels were similar in all females and younger masculinized females, but significantly lower in older masculinized females and males. Naïve males had significantly lower brain levels of both nonapeptides than males. In females and masculinized females, the concentrations of these neurohormones were age dependent: brain levels of AVT and IT were significantly lower in older females and older masculinized females, respectively.

Calculation of the AVT:IT ratios for the different groups gave the following values: masculinized females – 1:7 (12 months old) and 1:9 (18 months old), females – 1:1 (12 months old) and 1:2 (18 months old), naïve males – 1:3 and males – 1:2. The proportions of AVT and IT in masculinized females were markedly different from those in females and males. The AVT:IT ratio also differed between naïve males and males. In females and masculinized females, this ratio changed with age.

**Fig. 2.**
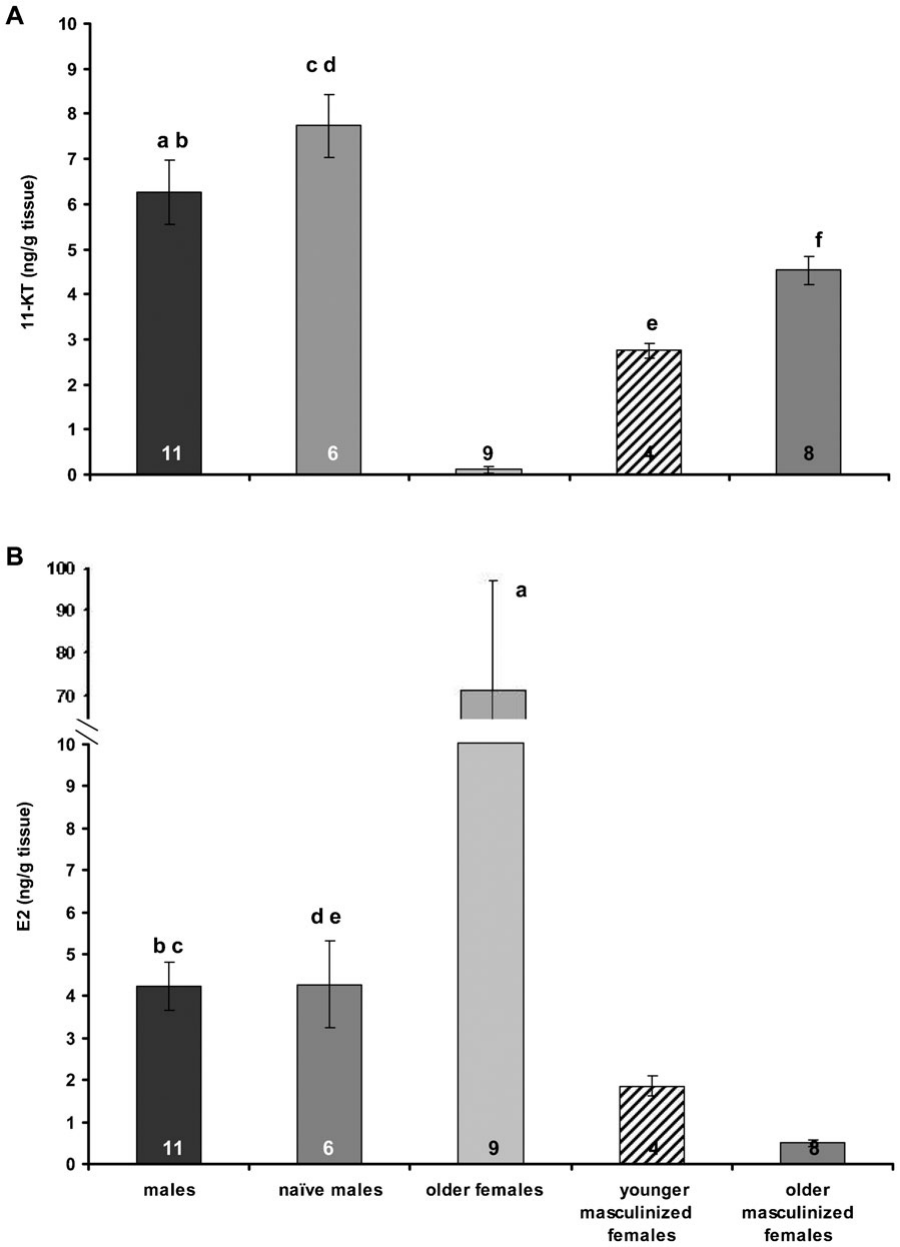
11-ketotestosterone (A) and 17-β estradiol (B) concentrations in gonads of males, naïve males, older females and younger and older masculinized females of *Poecilia sphenops*. Values are presented as means ± SEM. Statistical differences are indicated as: (A) a: *P*<0.001 vs older females, b: *P*<0.05 vs younger masculinized females, c: *P*<0.001 vs older females and younger masculinized females, d: *P*<0.01 vs older females and older masculinized females, e: *P*<0.01 vs older females, f: P<0.001 vs older females. (B) a: *P*<0.001 vs the others, b: *P*<0.05 vs younger masculinized females, c: *P*<0.001 vs older masculinized females, d: *P*<0.05 vs younger masculinized females, e: *P*<0.01 vs older masculinized females.

Concentrations of gonadal 11-KT (Fig. 2A) and E_2_ (Fig. 2B) were measured in younger and older masculinized females, females, naïve males and males. In females, 11-KT levels were low and E_2_ levels were high, whereas the opposite was found in all males. In all masculinized females, 11-KT levels were significantly higher than those in females, but lower than in all males. E_2_ levels were lowest in masculinized females. In naïve males, the 11-KT and E2 levels were similar to those of males. There were no significant differences in the GPI (gonopodial index) between males (18.47 ± 0.50), naïve males (18.97 ± 0.81) and younger (16.75 ± 0.4) or older (17.56 ± 0.6) masculinized females.

Pearson's correlation analysis (Table 1) showed no relationship between brain AVT and IT concentrations in females, masculinized females or males. However, a strong positive correlation (r = 0.71; *P*<0.001) was demonstrated in naïve males. Positive correlations were also identified between IT and E_2_ (r = 0.52; *P*<0.001) in older masculinized females, and between AVT and E_2_ in males (r = 0.43; *P*<0.01). Negative correlations were demonstrated between the AVT and 11-KT levels in naïve males (r = −0.50; *P*<0.01), older females (r = −0.54; *P*<0.001) and older masculinized females (r = −0.62; *P*<0.001).

**Table 1.**
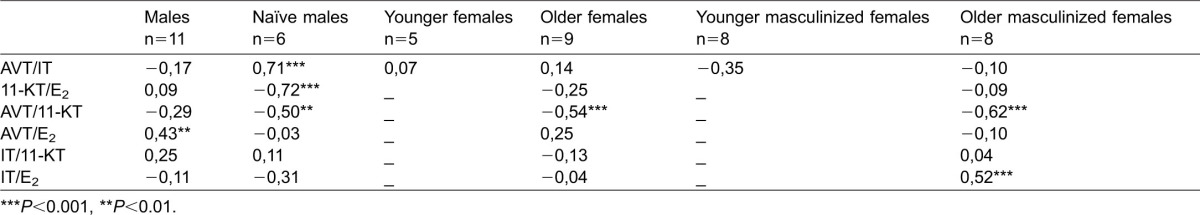
Pearson's correlation analysis

**Fig. 3.**
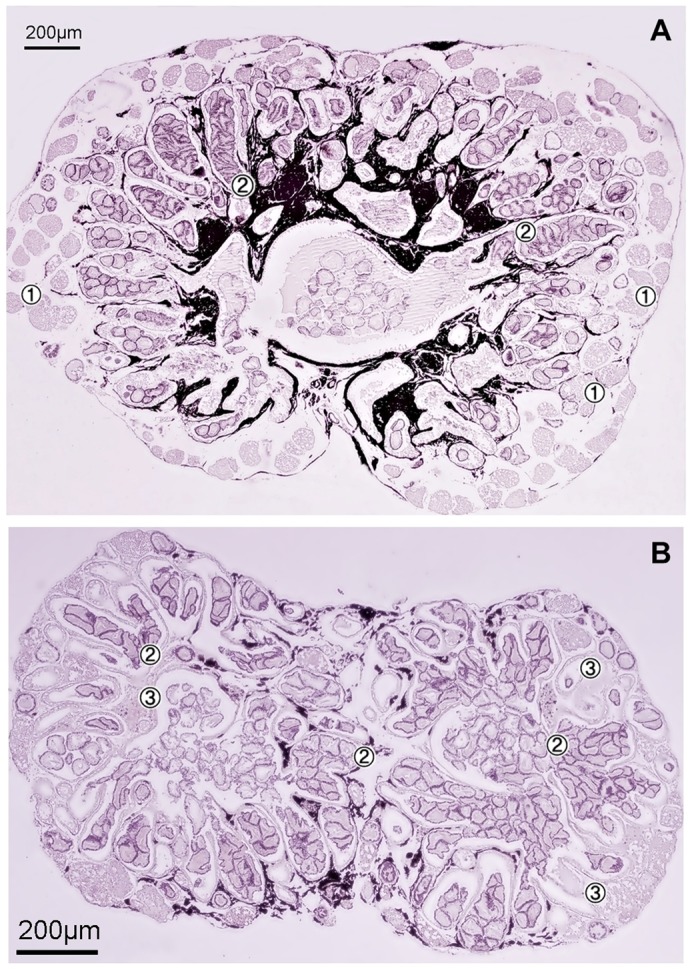
Transverse section of gonad of male (A) and masculinized female (B) of *Poecilia sphenops*. In male and masculinized female, germ cells are in all spermatogenic stages. Connective tissue constituted about 5% of both gonads. (A) Progressing stages of germ cells are distributed from the distal to proximal part. Spermatogonia (1) are restricted to the distal termini of the lobules. Spermatozeugmata (2) are regularly distributed within central part of the lobules. (B) Spermatocysts (spermatogenic cysts) (3) are irregularly organised. Large bundles of spermatozeugmata (2) are irregularly distributed.

**Fig. 4.**
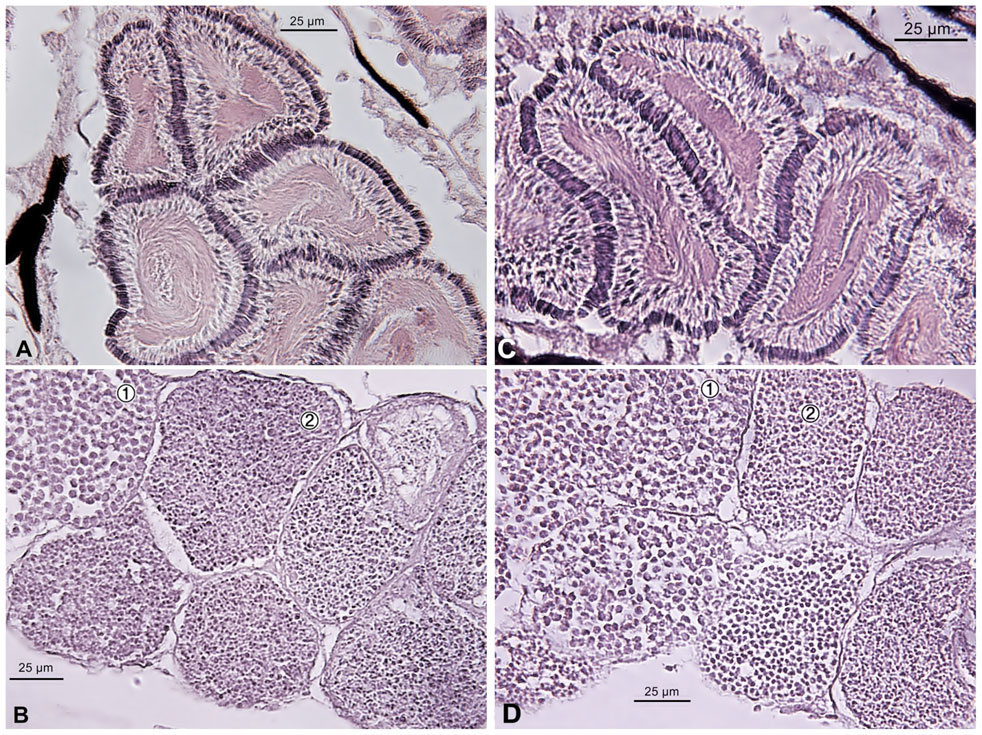
Transverse section of testis of male (A,B) and naïve male (C,D) of *Poecilia sphenops*. (A,C) spermatozeugmata, bundles of spermatozoa with heads pointing outwards and tails in the center; (B,D) regularly organised cysts with different stages of spermatogenesis: spermatocytes (1), spermatids (2).

Images of the gonads of females, males, naïve males and masculinized females were compared. The gonads of masculinized females were bigger and heavier than male testis (0.030 ± 0.004 *vs* 0.013 ± 0.002 g respectively, *P*<0.01). In contrast to testes, the masculinized individuals' gonads contained disorganized spermatocysts (spermatogenic cysts) and irregularly distributed large bundles of spermatozeugmata (Fig. 3A,B). The results of Experiment 4 showed that masculinized individuals were infertile. In naïve males, the microscopic structure of the gonads were similar to those of males (Fig. 4A–D).

## DISCUSSION

Responses of black molly females and males to the lack of mates in the group were different: masculinization and same-sex sexual behaviour, respectively. Our findings revealed that masculinization involves brain AVT and gonadal steroids, whereas same-sex sexual behaviour involves both brain nonapeptides, AVT and IT, but not sex steroids.

There is evident sexual dimorphism in brain AVT and IT concentrations in the black molly, just like that described previously in the three-spined stickleback (Kleszczyńska et al., 2012; Kleszczyńska and Kulczykowska, 2013). In female brains, irrespective of age, we measured significantly higher AVT and IT concentrations than in the brains of males. As was confirmed in younger and older masculinized females, the process of masculinization is apparently linked to a decrease in whole brain AVT levels towards values typical for males. A reduction in brain IT was also noted, but only in older masculinized females. The masculinization process appears to be associated not only with alterations in the concentrations of single nonapeptides, but also with the AVT:IT ratio in the brain. The activity of cells producing AVT and the abundance of its receptor transcript in the brain are sex-dependent and coupled with changes in sex phenotype in some fish species (Grober and Sunobe, 1996; Reavis and Grober, 1999; Godwin et al., 2000; Semsar and Godwin, 2003; Maruska et al., 2007; Lema et al., 2012). In the present study, we observed masculinization in younger or older individuals, but the factors responsible for the timing of the occurrence of this phenomenon remain unknown. We also demonstrated the influence of age on brain nonapeptide levels (Fig. 1A,B) which should be taken into consideration when planning future studies.

Sexually naïve males exhibiting gonopodial displays towards tank mates and copulation attempts had significantly lower levels of both AVT and IT than males not displaying such behaviour (Fig. 1A,B). The AVT:IT ratios were also different. This finding is the first indication of a relationship between brain nonapeptides and the occurrence of same-sex sexual behaviour in fish. There have been no other similar studies in lower vertebrates, but in humans, Swaab and Hofman (Swaab and Hofman, 1990) found an enlarged vasopressinergic subnucleus in the SCN (suprachiasmatic nuclei) of the hypothalamus of homosexual men, suggesting a link between the nonapeptide vasopressin, the human counterpart of vasotocin, and types of sexual attraction in humans. In general, little is known about neurohormonal mechanisms controlling mammalian sexual attraction, not to mention lower vertebrates. Besides indicating the involvement of nonapeptides AVT and IT in same-sex mating in fish, our data also suggest that the production of nonapeptides in the male brain is stimulated by sexual contact with females, because AVT and IT concentrations were significantly higher in female-experienced individuals than in sexually naïve ones (Fig. 1A,B).

Correlation analysis showed no relationship between brain levels of AVT and IT in females, masculinized females or males that do not display same-sex mating behaviour. This is not surprising because it is known that AVT and IT are synthesized independently in separate neurones in the POA (Holmqvist and Ekström, 1995; Saito et al., 2004). However, the finding of a strong positive correlation between brain AVT and IT levels in naïve males displaying same-sex mating was unexpected. Perhaps this suggests a direct functional connection between AVT- and IT-producing neurones (or a common regulatory mechanism) that is linked to specific behaviour or the lack of sexual experience of sexually naïve males. Although this observation is currently difficult to explain, it certainly warrants further study.

In this study, we also examined the involvement of gonadal steroids in the determination of phenotypic sex and sexual behaviour, and the relationship between these sex hormones and brain nonapeptides. Gonads are the main sources of circulating sex hormones (Kime, 1993) therefore the analysis of 11-KT and E_2_ in gonads is applicable when plasma collection is not possible or the plasma volume is low (Becker et al., 1992). This approach is also useful when a link between a local production of sex hormones and gonads' morphology is discussed as it is in this case. In black molly masculinized females, the levels of 11-KT were significantly higher and E_2_ significantly lower than in females. This result was not unexpected because a similar observation was made in the Hawaiian saddleback wrasse (*Thalassoma duperrey*), a protogynous reef species, (Nakamura et al., 1989) where sex hormones were measured in plasma. In addition, Cardwell and Liley (Cardwell and Liley, 1991a; Cardwell and Liley, 1991b) found an elevation of 11-KT and a decline in E_2_ in the plasma of stoplight parrotfish (*Sparisoma viride*) during a female-to-male sex change. It is also known that gonopodium formation depends on androgens (Ogino et al., 2004), and the stage of development of this organ should reflect changes in the androgen level. However, in black molly, 11-KT levels were considerably lower in masculinized females than in males, despite the fact that both have fully developed gonopodia. It should be noted that the macroscopic and microscopic structures of the gonads of males and masculinized females were markedly different. The masculinized individuals are steril (Experiment 4), cannot reproduce and cannot regress back into females. This maladaptive phenotype probably resulted from generations of domestic inbreeding.

On the other hand, it should be stressed that the microscopic structure of the gonads and sex steroid levels were identical in all males, irrespective of whether or not they displayed same-sex mating. Thus the occurrence of same-sex mating seems to be independent of the sex steroid status. A strong negative correlation between 11-KT and E_2_ levels was only observed in sexually naïve males, which suggests that an intrinsic negative feedback mechanism may operate in the gonads that is linked to a specific behavioural phenotype and/or the lack of sexual contact with females. In addition, the timing of the masculinization seemed to be related to the steroid concentration: the later this process occurred, the higher the 11-KT level and the lower the E_2_ level. However, the number of individuals examined is too small to draw any firm conclusions.

Given that the only way that social cue (the lack of a mate in this study) can affect the gonads and behaviour is via the brain, we suppose that neural mechanisms initiate socially-dependent phenotypic sex and behavioural changes in the black molly. Among several neuroendocrine pathways that could be engaged in the transduction of social signals, a likely candidate is the gonadotropic signalling pathway. A recent study by Kim et al. (Kim et al., 2012) showed that GnRH peptides play important roles in the regulation of the hypothalamic-pituitary-gonadal axis and probably control gonad development and sex change in the cinnamon clownfish (*Amphiprion melanopus*). It is probable that a GnRH-related cue is responsible for the increased 11-KT and decreased E_2_ levels detected in the gonads of black molly masculinized individuals. However, gonadotropic signalling is not the only neurohormonal mechanism that can stimulate phenotypic sex change and control the behavioural phenotype in fish. In the present study, we have shown that brain AVT is linked to socially dependent masculinization and changes in sexual behaviour of females. The male-like behaviour of masculinized individuals, i.e. movements of the gonopodium (“full swing”), courting and copulation attempts with females, is associated with a lower “male” brain AVT level. Because the brain nonapeptide levels were measured when the process of masculinization was complete (confirmed by the fully developed gonopodium and analysis of gonads), we can only speculate that the decrease in brain AVT concentration acts to trigger the phenotypic and behavioural changes. On the other hand, the male-like behaviour of masculinized females does not seem to be linked to the brain IT, because levels of this nonapeptide were similar in all females and younger masculinized individuals, and were lower only in older masculinized individuals that behave just the same as younger ones.

Previous studies have demonstrated relationships between AVT, androgen levels and sexual phenotypes in various fish species (see for example: Goodson and Bass, 2001; Semsar and Godwin, 2004; Oliveira et al., 2005; Aubin-Horth et al., 2007). The nature of the relationship between the GnRH- and AVT/IT-related signalling systems in black molly remains an open question. There is evidence that the GnRH and AVT pathways overlap in Teleostei. GnRH-synthesizing neurones are widely distributed in the POA hypothalamus region adjacent to the AVT-containing neurons in the brain of the green molly *Poecilia latipinna* (Batten et al., 1990). In the ventral POA of dwarf gourami (*Colisa lalia*), IT-ir neurons are also immunoreactive to GnRH (Maejima et al., 1994). The significant correlation between brain nonapeptides and gonadal steroids that we have identified in black molly is a strong indication of cross talk between these hormonal systems. Interestingly, the character of this interaction, be it positive or negative, depended on the sexual phenotype of individuals. Because a correlation refers to any relationship between two variables and does not identify the cause or the effect, two different scenarios may be considered: (1) GnRH stimulates AVT/IT neurones directly via neural pathways and/or via sex steroids, and (2) AVT/IT triggers GnRH-related cues, i.e. sex steroid production. Both of these scenarios receive support in the literature (Saito et al., 2003; Rodríguez and Specker, 1991).

The biological context of the occurrence of masculinization and same-sex mating in black molly deserves some comment. In the present study, only females from an overcrowded group underwent masculinization. This suggests that high-density stress plays some role in promoting the process of masculinization. In some fish species, cortisol or its metabolites produced and secreted during stress cause masculinization of females or induce sex change (Hattori et al., 2009; Grillitsch et al., 2010), probably because the molecular structure of glucocorticoids and 11-KT are similar. As demonstrated during high temperature-induced masculinization in several fish species, cortisol can also promote 11-KT production in gonads (Fernandino et al., 2012). However, in the case of the black molly, none of the females showed any of the typical signs of stress or discomfort such as aggressiveness, going into hiding or avoidance behaviour. Therefore, we propose that other cues, such as pheromones or visual stimulation, may be engaged in the masculinization process. Recently, Mangiamele et al. (Mangiamele et al., 2013) showed that exposure of male goldfish to androstenedione, a pheromone released by males and females, inhibits social approaches towards other males and increases AVT gene expression in the POA. There is growing evidence on female responses to pheromones (Corkum et al., 2008) and it seems likely that these chemical cues play a pivotal role in causing black molly females to change sexual phenotype. An abundance of E_2_ and its derivatives released by many females and/or a lack of male chemical markers (11-KT and/or its derivatives) may influence the proportion of sex steroids in the gonads, which results in masculinization. The very low E_2_ levels measured in the gonads of masculinized individuals, that were even below “male” values, seems to confirm this. Vision plays a pivotal role in the reproductive behaviour of many fish species, especially in the *Poeciliidae* (Kodric-Brown and Nicoletto, 2001; Plath et al., 2005; Tobler et al., 2006), so a second candidate for the signal triggering masculinization in the black molly is visual stimulation. In the present case, the visual stimulus is the perception of many females in close proximity. Recently, Amorim et al. (Amorim et al., 2013) demonstrated that visual courtship signal is relevant in mate choice in the painted goby (*Pomatoschistus pictus*). In a similar way, a lack of sensory cues from females and a presence of visual or chemical signals from males, may promote the same-sex sexual behaviour in naïve males. Both of the above scenarios, pheromones- and vision-related, are possible and further research is required to establish which cue is critical in initiating masculinization and same-sex sexual behaviour in black molly females and males.

In summary, our findings show that brain AVT and gonadal steroids are engaged in masculinization of the black molly females. On the other hand, the same-sex sexual behaviour of sexually naïve males is linked to brain AVT and IT levels, but does not involve gonadal steroids. The occurrence of masculinized individuals that display typical male behaviour allows females to gain sexual experience in situations where there is a dearth of natural sexual partners in the group. Same-sex mating appears in mono-sex groups of sexually naïve males or sexually experienced males, with only the latter showing aggressive behaviour. In lower vertebrates, male-male sexual attraction is usually considered in the context of dominance rank, but in our model species, there is no dominance hierarchy. It should be noted that males have only a passing predisposition to same-sex mating; when introduced into a mixed-sex group, sexually experienced or naïve males court and copulate only with females. In non-human animals, most males that engage in same-sex encounters also mate with females (Bailey and Zuk, 2009). In conclusion, we have demonstrated, for the first time in lower vertebrates, that brain AVT and IT may be involved in socially controlled sexual acts that are not directly linked with procreation and reproductive success.

## MATERIALS AND METHODS

### Animals and experimental design

The viviparous black molly (*Poecilia sphenops*), a well-known aquarium fish, is a short-finned hybrid of *Poecilia latipinna*, the sailfin molly that is not present in nature. In this year-round breeder, fertilization is internal. In both *P. latipinna* and *P. sphenops*, male mate choice copying has been found (Gabor and Aspbury, 2008). A laboratory stock of black mollies (males and females) was bred and maintained at a temperature of 27 ± 1°C in water of 0.2 ppt salinity with a 12L:12D photoperiod in aerated aquaria at the Institute of Oceanology PAN (IO PAN Sopot, Poland), where all experiments were carried out. The fish were fed commercial Ichtio-vit flake food, and frozen mosquito and brine shrimp larvae. In each experiment, the fish were sampled 3 h after the onset of the light period. The following experiments were performed using fish from the laboratory stock:

#### Experiment 1 – females that do not undergo masculinization and males that do not display same-sex sexual behaviour

Adult sexually mature females of 9–10 months of age were randomly selected from the mixed sex laboratory stock of black mollies. At the beginning of the experiment all females were of equivalent reproductive state. When kept at low density (5 fish in an aquarium of 50 L) none of these females underwent masculinization. The fish behaved indifferently towards each other. The experiment was repeated 6 times and provided 30 females for further analyses. The fish were sampled at the ages of 12 and 18 months to be comparable to females which underwent masculization at the ages of 12 and 18 months, respectively (Table 2).

**Table 2.**
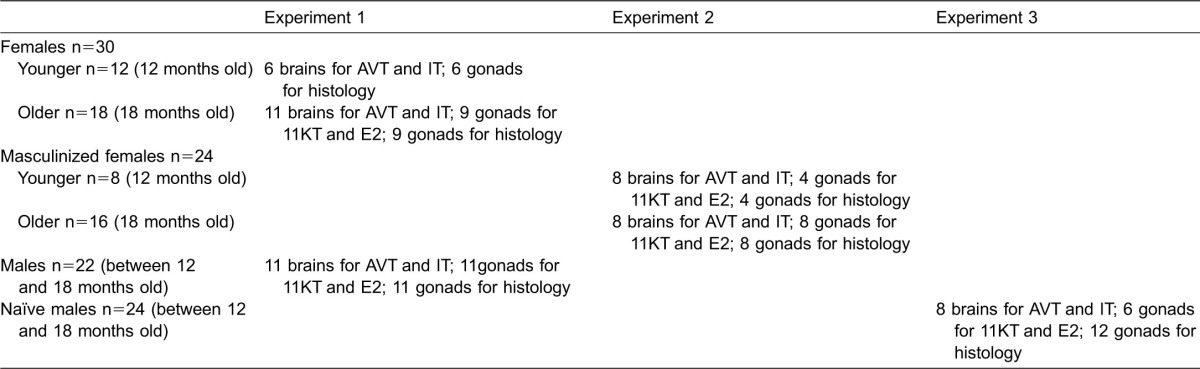
Animals and sampling protocols in Experiments 1–3

Two adult males of 12–18 months of age selected from the stock were kept with two adult females of the same age in a 50-L aquarium. The males directed their sexual interest only towards the females and showed a mating preference for large individuals. They courted females, performing a “full swing” (gonopodium swung forward 180° from its resting position), and attempted to copulate with them. The experiment was repeated 11 times and provided 22 males for further analyses. The males were aged between 12 and 18 months when sampled (Table 2).

#### Experiment 2 – masculinized females

Adult females of 9–10 months of age were randomly selected from the mixed sex laboratory stock of black mollies to constitute female-only groups. In all groups of females kept at high density (15 females in an aquarium of 50 L) only one individual underwent masculinization. The masculinization was completed in individuals at the age of 12 or 18 months. Therefore masculinized females were divided up into two groups (younger and older) and compared with females from Experiment 1 sampled at the ages of 12 and 18 months, respectively. At the start of the masculinization process an individual with a barely visible gonopodium behaved indifferently towards conspecifics, but later on when its gonopodium had developed in size, it performed a “half swing” (gonopodium swung forward 90° from its resting position) towards females. Individuals with a fully developed gonopodium displayed a “full swing” and attempted to copulate with females. Full development of the gonopodium occurred over about 3 months. The masculinization process was irreversible and produced individuals with distinct features. The masculinized individuals were as big as females, had a larger dorsal fin than females and a gonopodium of the same size as that of males and changed morphology of gonads. In this study, only individuals with a fully developed gonopodium were sampled. The experiment was repeated 24 times and provided 24 masculinized females for further analyses (Table 2).

#### Experiment 3 – triggering same-sex sexual behaviour

Since just after hatching, juveniles were kept in a 40-L aquarium for six months. The males were removed as soon as their gonopodium became visible. Males of about the same age (12–18 months) and body size (44.7 ± 1.4 mm and 0.96 ± 0.13 g) were put into two 50-L aquaria of 12 individuals per aquarium. All of these female-inexperienced males (naïve males) exhibited same-sex sexual behaviour manifested by gonopodial displays towards same-sex tank mates and copulation attempts. When the group was divided between six 50-L aquaria of 4 individuals per aquarium gonopodial displays and copulation attempts had become even more pronounced. It should be noted that female-experienced males in male-only groups also displayed same-sex sexual behaviour. However, in this situation, the female-experienced males were always aggressive towards each other, whereas the sexually naïve males were not. In this study, we sampled and analyzed only the naïve males to avoid any impact of aggressive behaviour on the results (Backström and Winberg, 2009; Lema and Nevitt, 2004; Iwata et al., 2010). The experiment provided 24 sexually naïve males displaying same-sex sexual behaviour. The males aged between 12 and 18 months were sampled from six 50-L aquaria for further analyses (Table 2).

#### Experiment 4 – determining the fertility of masculinized individuals

One masculinized female at the age of 12 or 18 months and two unfertilized mature females of between 12 and 18 months old from the low-density female-only group (Experiment 1) were put together in a 50-L aquarium for 3 months. The masculinized female was then removed and the females were observed for the next 3 months. This experiment was repeated many times, but offspring were never produced. In comparison, when one adult male from the stock (aged between 12 and 18 months with a fully developed gonopodium) and two unfertilized mature females from the low-density female-only group were put together in a 50-L aquarium for 3 months, this usually resulted in the appearance of progeny.

### Tissue sampling

Fish selected for further analyses were anaesthetized by immersion in MS 222 (tricaine methanesulphonate; Sigma-Aldrich, USA) solution in water (50 mg L^−1^), their spinal cords were sectioned and whole brain together with pituitary and gonads removed (Table 2). The brains were weighed and stored at −70°C prior to AVT and IT analyses. The gonads were weighed, subjected to morphological examination and then either stored at −70°C prior to E_2_ and 11-KT measurement or immediately fixed in 4% formalin solution for histology. The gonopodial index (GPI) was calculated by expressing the gonopodial length as a percentage of the total body length.

The ranges of total length and weight of individuals were as follows: males – 44.7 ± 1.4 mm and 0.96 ± 0.13 g; younger females – 40.7 ± 0.5 mm and 1.00 ± 0.07 g; older females – 54.9 ± 0.5 mm and 3.44 ± 0.61 g; younger masculinized females – 47.8 ± 1.2 mm and 1.62 ± 0.17 g; older masculinized females – 51.4 ± 2.4 mm and 2.35 ± 0.39 g. The older females and masculinized females were significantly bigger than younger ones (p<0.01) and younger masculinized females were significantly bigger than younger females (p<0.01).

All experiments complied with EC Directive 2010/63/EU for animal experiments and with the guidelines of the Local Ethics Committee on Animal Experimentation.

### Chemical analyses

#### Arginine vasotocin and isotocin

The AVT and IT concentrations in fish brains were measured using the procedure described by Kulczykowska (Kulczykowska, 1995). Briefly, the brains were individually sonicated in 1 mL of 0.25% acetic acid using a Microson XL 2000 (Misonix Inc, NY, USA) and the sonicates were placed in a boiling water bath for 3.5 min. The extracts were cooled on ice and then centrifuged (20,000 ***g***, 20 min, 4°C). The supernatants were removed and loaded onto solid phase extraction (SPE) columns (Bakerbond C18, 100 mg mL^−1^, JT Baker, USA) that had been pre-conditioned with methanol followed by water. Impurities were washed from the loaded columns with 1 mL of 5% acetic acid followed by 1 mL of water and 1 mL of 5% methanol. Peptides were eluted with 2 mL of an ethanol:HCl mixture (2000:1) and this elution was repeated once. The pooled eluates were evaporated to dryness in a TurboVap LV^TM^ (Caliper Life Science, USA). Using the procedure described by Kulczykowska (Kulczykowska, 1995), the efficiency of AVT and IT recovery following SPE extraction was estimated as 89–94% and 92–95%, respectively.

High performance liquid chromatographic (HPLC) analyses were performed using a 1200 series Quaternary HPLC system (Agilent Technologies, USA) with a diode array detector. Chromatographic separation of peptides was carried out on an Ultrasphere ODS column (250 mm × 4.6 mm; 5 µm) following a pre-column (45 × 4.6 mm I.D.) comprised of the same material (both columns obtained from Beckman Coulter, USA). Separation of peptides was achieved with a linear gradient system consisting of 20–40% solvent B (0.1% trifluoroacetic acid (TFA) in acetonitrile:water, 3:1) in solvent A (0.1% TFA in water) over 20 min. The column temperature was 20°C and flow rate 1 mL min^−1^. UV detection was performed at 215 nm. The equation of the AVT calibration curve was y = 0.795x (R^2^ = 0.999), and that for IT, y = 0.864x (R^2^ = 0.997). The limits of detection (LOD) of AVT and IT were evaluated at a signal-to-noise ratio of 3:1 as 15 and 18 pmol mL^−1^, respectively. The intra-day precision of the method, expressed as relative standard deviation (RSD), was within the ranges 3.6–6.1% and 4.4–5.9% for AVT and IT, respectively. The concentrations of these brain neurohormones were expressed as ng per g wet weight of tissue.

#### Gonadal steroids

Gonads were individually sonicated in 0.5 mL of phosphate buffer (0.05 M, pH 7.4) supplemented with sodium azide (NaN_3_) using a Microson^TM^ XL 2000 (USA). The sonicates were centrifuged at 20,000 ***g*** for 20 min at 4°C and the supernatants stored at −70°C prior to the analysis of E_2_ and 11-KT levels.

##### 11-ketotestosterone

The 11-KT concentration in gonad organic extracts was determined using a Cayman's competitive enzyme immunoassay (EIA) kit (Ann Arbor, MI, USA). Gonad supernatants (250 µL) were extracted with 1 mL of ethyl acetate/hexane (50:50 v/v) according to the method recommended in the EIA kit protocol. The samples were then held at −20°C for 15 min to separate the layers. The ethyl/hexane layer was decanted into a glass tube and evaporated under a stream of nitrogen. This procedure was repeated three times. Dried extracts were stored at −20°C prior to analysis. The recovery rate of the extraction was between 98–115%. Extracts were dissolved in 0.5 mL of EIA buffer and samples of 50 µL were taken for analysis. 11-KT-acetylocholinesterase (AChE) conjugate was used as a tracer. A standard curve was prepared using eight standard dilutions of 11-KT: 0.78, 1.56, 3.13, 6.25, 12.5, 25, 50 and 100 pg mL^−1^. The assay was conducted in microplates according to the EIA kit manufacturer's instructions with slight modifications. Microplates were gently shaken for 15 min, incubated for 18 hours at 4°C and washed with buffer using a HydroFlex strip-washer (Tecan, Austria). The plates were then developed by shaking with Ellman's reagent in the dark for 90 min and absorbance at 412 nm was read using a Sunrise Absorbance Reader (Tecan, Austria). All samples were assayed in duplicate. The detection limit of the assay was 13 pg mL^−1^. The intra-assay coefficient of variation was 0.8%. The inter-assay coefficient of variation was 7.8%. The concentration of 11-KT was expressed as ng per g wet weight of tissue.

##### 17β-estradiol

The E_2_ concentration in gonad organic extracts was determined using a Spectria Estradiol radioimmunoassay (RIA) kit (Orion Diagnostica, Finland). Gonad supernatants (200 µL) were extracted with 1.6 mL of ethyl ether according to a modification of the method of Mori and Kano (Mori and Kano, 1984). Samples were vortexed for 1 min at 350 ***g***, for 30 min at 35 ***g*** and then held at −20°C for 30 min to separate the layers. The ethyl ether layer was decanted into a glass tube and evaporated under a stream of nitrogen. Dried extracts were stored at −20°C prior to analysis. The recovery rate of the extraction was between 86–109%. Extracts were dissolved in phosphate buffer (0.05 M, pH 7.4) supplemented with NaN_3_ and samples of 100 µL were taken for RIA analysis. Iodinated E_2_ with ^125^I was used as a tracer. A standard curve was prepared using six standard dilutions of 50, 150, 500, 1500, 5000 and 15,000 pmol L^−1^. The assay was conducted in RIA tubes according to the kit manufacturer's instructions with slight modifications. The samples were added to tubes that had been pre-coated with polyclonal anti-rabbit antiserum. After vortexing for 10 seconds, the tubes were incubated for 2 hours at 37°C, decanted, washed with 1 mL of 6× concentrated Tween 20 solution and decanted again. Radioactivity in each tube was measured for 1 min using a Wallac Wizard 1470 gamma counter (Perkin Elmer Life Science, USA). All samples were assayed in duplicate. The detection limit of the assay was 37 pmol L^−1^. The intra-assay coefficient of variation was 6.5%. The inter-assay variation was not determined because all samples were measured in the same assay. The concentration of E_2_ was expressed as ng per g wet weight of tissue.

### Histological analysis of gonads

Whole gonads were fixed in 4% formalin solution, dehydrated in ethanol and embedded in paraffin. Sections of 5 µm were cut using a RM2245 microtome (Leica Microsystems GmbH, Germany) and stained with Mayer's haematoxylin and eosin. Slides prepared from each gonad were examined with a Leica HI1210 light microscope (Leica Microsystems GmbH, Germany). The developmental stage of testes was determined according to the classification worked out for oviparous teleosts by Grier (Grier, 1981) and modified by Kinnberg et al. (Kinnberg et al., 2003), while the developmental stage of oocytes was determined according to the classification worked out for the guppy, *Poecilia reticulate*, by Takano (Takano, 1964) and modified by Koya (Koya et al., 1998).

### Statistical analyses

Statistical analyses of data were carried out using Statistica 5.1 software. Values are presented as means ± SEM. For multiple comparisons of hormone levels, analysis of variance (one-way ANOVA) was performed followed by post hoc tests (Tukey's test for equal numbers of cases or Spjotvoll and Stoline's test for unequal numbers of cases). Pearson's correlation coefficient was used to measure the strength and direction of the linear relationship between hormones (all combinations). Student's t-test was used to detect differences in GPI. Significance was accepted at *P*<0.05.
